# The complete mitogenome of the *Amolops jinjiangensis* (Anura: Ranidae)

**DOI:** 10.1080/23802359.2021.1960915

**Published:** 2021-08-02

**Authors:** Ziwen Wang, Jun Ping, Yun Xia, Xiaomao Zeng, Jianghong Ran

**Affiliations:** a Chengdu Institute of Biology, Chinese Academy of Sciences, Chengdu, China; b Key Laboratory of Bio-Resources and Eco-Environment of Ministry of Education, College of Life Sciences, Sichuan University, Chengdu, China; c University of Chinese Academy of Sciences, Beijing, China

**Keywords:** *Amolops jinjiangensis*, mitochondrial genome, RAD-Seq

## Abstract

We describe the mitochondrial genome sequence of a torrent frog, *Amolops jinjiangensis*. The mitogenome was extracted and assembled for the first time by restriction site-associated DNA sequencing (RAD-seq). The total length is 17,780 bp, containing 13 protein-coding genes (PCGs), two ribosomal RNA genes, 22 transfer RNA genes, and one control region. The gene rearrangement was detected as the W-O_L_-ANCY gene cluster which consisted with several published *Amolops* mitogenomes. The phylogenetic tree was constructed based on 13 protein-coding genes of *A. jinjiangensis* and 11 closely related species by Bayesian analyses.

*Amolops jinjiangensis*, one of the torrent frogs that inhabit rapid-flowing mountain streams or waterfalls, is distributed along the Jinsha River basin in the Hengduan Mountains of southwestern China (Su et al. 1986; Frost 2021). The previous studies had phylogenetic inferences based on the partial mitochondrial sequences, i.e. *COI* and *Cyt-b*, but the phylogenetic relationship and position of *A. jinjiangensis* were remaining controversial (Lu et al. 2014; Lyu et al. 2019; Zeng et al. 2020). In this study, we identified complete mitochondrial genomes of the *A. jinjiangensis* by using restriction site-associated DNA sequencing (RAD-seq) to gain additional molecular information and contribute to better understanding of the evolutionary aspects of this frog taxon.

The female adult *A. jinjiangensis* collected from the type locality, Benzilan Town of Deqin County, Yunnan Province in China (N28°13′57.21″, E99°14′43.38″, 2704 m) in July 2015, and the voucher specimen (CIB-XM6120) was deposited in the Chengdu Institute of Biology, Chinese Academy of Sciences (http://www.cib.ac.cn/, contact Xiaomao Zeng and zengxm@cib.ac.cn). The muscle tissue isolated from the fresh specimen was preserved in 95% ethanol at −20 °C until use. Genomic DNA was extracted using Genomic DNA Kit (Sangon Biotech, Shanghai) and the single-digest restriction site-associated DNA sequencing (RAD-Seq) library preparation was carried out following the protocol of Baird et al. (2008) by the Novogene (Beijing, China). Briefly, genomic DNA was digested using restriction enzymes *EcoRI*, and cut-site fragments were sequenced by the Illumina Hiseq-PE150 platform. A total of 9,272,001,300 clean base (bp) was obtained by removing the contaminant sequences and the low-quality regions from raw data. The available clean data of *A. jinjiangensis* was directly assembled a complete mitogenome by MIRA v4.0.2 and MITObim v1.9.1 (https://github.com/chrishah/MITObim; Hahn et al. 2013). We implement the iterations 60 times and take closely related *A. granulosus* mitogenome (NC_044901.1) as the reference information. Finally, the assembled mitogenome sequence was annotated by using MITOS web server (http://mitos2.bioinf.uni-leipzig.de/index.py; Bernt et al. 2013) and the MPI-MP CHLOROBOX tools (https://chlorobox.mpimp-golm.mpg.de/OGDraw.html; Greiner et al. 2019).

The whole sequence of the *A. jinjiangensis* mtDNA was deposited to the GenBank DNA databases under accession number MZ292455. The complete mitogenome of *A. jinjiangensis* was 17,780 bp in length and it contained the 37 typical genes: two ribosomal RNAs, 22 transfer RNAs (tRNAs), 13 protein-coding genes (PCGs), and one putative control region (D-loop). The length of 12S rRNA was 932 bp, and of 16S rRNA was 1586 bp, while 22 tRNAs ranged from 65 to 73 bp, which were similar to other *Amolops* species. Overall nucleotides base composition of the complete mtDNA is 28.03% for A, 15.05% for G, 28.87% for C, 28.05% for T, with a higher A + T content (56.08%). The gene rearrangement was detected as the W-O_L_-ANCY gene cluster in the mitochondrial genome of *A. jinjiangensis*, which is consistent with five published *Amolops* mitogenomes including *A. mantzorum*, *A. loloensis*, *A. tuberodepressus*, *A. chuanganensis*, and *A. granulosus*, but different from the other three *Amolops* species, i.e. *A. ricketti*, *A. wuyiensis* and *A. hongkongensis*.

The phylogenetic tree (Figure 1) was constructed based on the 13 PCGs of *A. jinjiangensis* and 11 closely related species. The best-fit nucleotide substitution models were determined using Partitionfinder 2.1.1 (Lanfear et al. 2017). Bayesian analyses were conducted using MrBayes 3.2.7 with the Marko chain Monte Carlo (MCMC) for 20,000,000 generations and 1000 sampled generations (Ronquist et al. 2012). The phylogenetic tree indicated that the mitogenome of *A. jinjiangensis* and *A. loloensis* clustered together, which supported the phylogenetic inferences estimated by Lyu et al. (2019) and Zeng et al. (2020) with samples also from Deqin County in Yunnan Province, but conflicted with those from Zhongdian County in Yunnan Province (Lu et al. 2014). Furthermore, our analyses recovered a sister taxa relationship between *A. jinjiangensis + A. loloensis* and *A. mantzorum + A. tuberodepressus.* Although there are no enough mitogenomes of *Amolops* to analyze phylogenetically, and more information about related species could be useful for a more detailed study of mitogenome evolution and phylogenetic relationships in *Amolops*.

**Figure 1.**
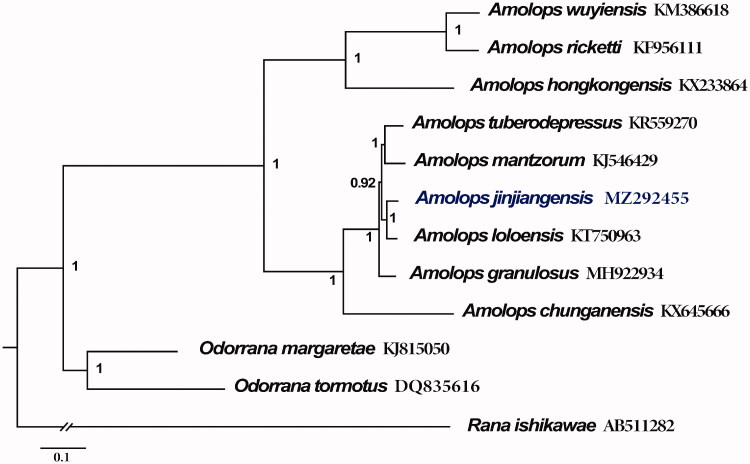
phylogenetic tree based on the concatenated nucleotide sequences of 13 PCGs from 12 species constructed with Bayesian inference (BI). For the BI tree, *Rana ishikawae* (AB511282) was used as outgroup. Bayesian posterior probabilities are shown near the nodes. The GenBank accession numbers of all species are shown.

## Data Availability

The genome sequence data that support the findings of this study are openly available in GenBank of NCBI at (https://www.ncbi.nlm.nih.gov/) under the accession no. MZ292455. The associated BioProject, SRA, and Bio-Sample numbers are PRJNA741045, SRR14901856, and SAMN19843588, respectively.
